# Texas State, Williamson County, Travis County, East Texas, East Line, and North Texas Med. Societies

**Published:** 1888-01

**Authors:** 


### TEXAS STATE MEDICAL ASSOCIATION.

The Chairman of the Committee on Revision of the Constitution and By-Laws, Dr. R. P. Talley, of Belton, requests us to ask members to send to him such suggestions with regard to the proposed new Constitution and By-Laws as submitted at last meeting and published in the Transactions, as may occur to them. It will be remembered that the New Constitution and By-Laws as drafted by the Committee, and as it appears in the Transactions, will come up for adoption on the first day of the coming meeting; and as it is a matter of much importance, the Committee desire the views of members on the subject.

WILLIAMSON COUNTY MEDICAL SOCIETY, 2nd annual meeting, January 1888. Officers for the year: President, Dr. J. E. Walker, Vice-Presidents, Dr. W. G. Pettus, Dr. H. N. Graves; Secretary, Dr. C. C. Black, of Round Rock.

TRAVIS COUNTY MEDICAL SOCIETY met Thursday, January 4 Dr. Florence E. Collins resigned as Secretary, and Dr. T. J. Bennett, the Secretary of the Austin District Medical Association, was unanimously elected. It was agreed to have a called meeting between each monthly meeting; and to infuse new life into the Society, it was determined to present at each meeting a subject for discussion, whether there were any papers or not, and to have one member appointed to open this discussion. Subject for discussion at meeting Jan. 19th inst., "Acute Pulmonic Inflammation in Children," Dr. Q. C. Smith to open discussion; Dr. Litten, alternate.

---

THE EAST TEXAS MEDICAL SOCIETY met at Jacksonville 1st Thursday in January inst., but owing to a misunderstanding as to date, the attendance was slim. This society numbers fifty active members, and they ride from five to fifty miles to attend the meetings.

---

THE EAST LINE MEDICAL SOCIETY meets at Sulphur Springs on 13th (or 3d ?) of February prox. A large meeting is expected, and some good papers. Dr. Paine will be there by invitation.

---

TEXAS PAPERS APPRECIATED ABROAD. WE have had a request from a publishing house in New York for a copy of the JOURNAL containing Dr. Beall's paper on "Uterine Fibroma Complicating Delivery," and from a physician in Kansas for one containing Dr. Paine's "Three Years with Electricity."

---

NORTH TEXAS MEDICAL SOCIETY held last meeting at Gainesville. Large attendance. The promise of "a full report" did not materialize, sorry to say.

PHYSICIANS' MUTUAL BENEFIT ASSOCIATION OF TEXAS. Assessment No. 5, has been issued for the benefit of Certificate No. 25, which was held by Dr. S. F. Starley, an account of whose death will be found in this issue. There were, at the date of his death,

Dec. 19, 1887, one hundred and sixty members in good standing, supposing that none forfeit on the payment of the dues for 1888 now due. Nine failed to pay assessment No. 4, and are dropped.

